# The growth and approximation for an analytic function represented by Laplace–Stieltjes transforms with generalized order converging in the half plane

**DOI:** 10.1186/s13660-018-1783-y

**Published:** 2018-07-24

**Authors:** Hong Yan Xu, Hua Wang

**Affiliations:** Department of Informatics and Engineering, Jingdezhen Ceramic Institute, Jingdezhen, China

**Keywords:** 44A10, 30D15, Approximation, Laplace–Stieltjes transform, Half plane, Error

## Abstract

By utilizing the concept of generalized order, we investigate the growth of Laplace–Stieltjes transform converging in the half plane and obtain one equivalence theorem concerning the generalized order of Laplace–Stieltjes transforms. Besides, we also study the problem on the approximation of this Laplace–Stieltjes transform and give some results about the generalized order, the error, and the coefficients of Laplace–Stieltjes transforms. Our results are extension and improvement of the previous theorems given by Luo and Kong, Singhal, and Srivastava.

## Introduction

Laplace–Stieltjes transforms

1 $$ F(s)= \int_{0}^{+\infty}e^{sx}\,d\alpha(x),\quad s= \sigma+it, $$

where $\alpha(x)$ is a bounded variation on any finite interval $[0,Y]$ ($0< Y<+\infty$), and *σ* and *t* are real variables, as we know, if $\alpha(t)$ is absolutely continuous, then $F(s)$ becomes the classical Laplace integral of the form

2 $$ F(s)= \int_{0}^{\infty}e^{st}g(t)\,dt. $$

If $\alpha(t)$ is a step-function and satisfies

$$\alpha(x)= \textstyle\begin{cases} a_{1}+a_{2}+\cdots+a_{n}, &\lambda_{n}< x< \lambda_{n+1};\\ 0, &0\leq x< \lambda_{1};\\ \frac{\alpha(x+)+\alpha(x-)}{2},&x>0, \end{cases} $$

where the sequence $\{\lambda_{n}\}_{0}^{\infty}$ satisfies

3 $$ 0=\lambda_{1}< \lambda_{2}< \lambda_{3}< \cdots< \lambda_{n}\uparrow+\infty, $$

thus $F(s)$ becomes a Dirichlet series

4 $$ F(s)=\sum_{n=1}^{\infty}a_{n}e^{\lambda_{n}s},\quad s=\sigma+it. $$

(*σ*, *t* are real variables), $a_{n}$ are nonzero complex numbers. Obviously, if $\alpha(t)$ is an increasing continuous function which is not absolutely continuous, then the integral (1) defines a class of functions $F(s)$ which cannot be expressed either in the form (2) or (4).

Let a sequence $\{\lambda_{n}\}_{n=1}^{\infty}$ satisfy (3), and

5 $$ \limsup_{n\rightarrow+\infty}(\lambda_{n+1}- \lambda_{n})=h< +\infty ,\qquad \limsup_{n\rightarrow+\infty} \frac{ n}{\lambda_{n}}=D< \infty. $$

Set

$$A_{n}^{*}=\sup_{\lambda_{n}< x\leq \lambda_{n+1},-\infty< t< +\infty} \biggl\vert \int_{\lambda _{n}}^{x}e^{ity}\,d\alpha(y) \biggr\vert , $$

if

6 $$ \limsup_{n\rightarrow+\infty}\frac{\log A_{n}^{\ast}}{\lambda_{n}}=0, $$

it is easy to get $\sigma_{u}^{F}=0$, that is, $F(s)$ is analytic in the left half plane; if

7 $$ \limsup_{n\rightarrow+\infty}\frac{\log A_{n}^{\ast}}{\lambda _{n}}=-\infty , $$

it follows that $\sigma_{u}^{F}=+\infty $, that is, $F(s)$ is analytic in the whole plane. For convenience, we use $\overline{L}_{\beta}$ to be a class of all the functions $F(s)$ of the form (1) which are analytic in the half plane $\Re s<\beta$ ($-\infty<\beta<\infty$) and the sequence $\{\lambda_{n}\}$ satisfies (3) and (5); $L_{0}$ to be the class of all the functions $F(s)$ of the form (1) which are analytic in the half plane $\Re s<0$ and the sequence $\{\lambda_{n}\}$ satisfies (3), (5), and (6); and $L_{\infty}$ to be the class of all the functions $F(s)$ of the form (1) which are analytic in the whole plane $\Re s<+\infty$ and the sequence $\{ \lambda_{n}\}$ satisfies (3), (5), and (7). Thus, if $-\infty<\beta<0$ and $F(s)\in \overline{L}_{\beta}$, then $F(s)\in L_{0}$.

In 1963, Yu [26] first proved the Valiron–Knopp–Bohr formula of the associated abscissas of bounded convergence, absolute convergence, and uniform convergence of Laplace–Stieltjes transform. Moreover, Yu [26] also estimated the growth of the maximal molecule $M_{u}(\sigma,F)$, the maximal term $\mu(\sigma ,F)$, by introducing the concepts of the order of $F(s)$, and investigated the singular direction–Borel line of entire functions represented by Laplace–Stieltjes transforms converging in the whole complex plane. After his wonderful works, considerable attention has been paid to the value distribution and the growth of analytic functions represented by Laplace–Stieltjes transforms converging in the whole plane or the half plane (see [1, 3, 4, 6–8, 11–15, 18–25, 27]).

Set

$$\begin{aligned} &\mu(\sigma,F)=\max_{n\in \mathbb{N}}\bigl\{ A_{n}^{*}e^{\lambda_{n}\sigma} \bigr\} \quad(\sigma< 0) , \\ & M_{u}(\sigma,F)=\sup _{0< x< +\infty,-\infty< t< +\infty} \biggl\vert \int _{0}^{x}e^{(\sigma+it)y}\,d\alpha(y) \biggr\vert \quad(\sigma< 0). \end{aligned} $$

For $F(s)\in L_{0}$, in view of $M_{u}(\sigma,F)\rightarrow+\infty$ as $\sigma\rightarrow0^{-}$, the concepts of order and type can be usually used in estimating the growth of $F(s)$ precisely.

### Definition 1.1

If Laplace–Stieltjes transform (1) satisfies $\sigma_{u}^{F}=0$ and

$$\limsup_{\sigma\rightarrow0^{-}}\frac{\log^{+}\log^{+}M_{u}(\sigma ,F)}{-\log(-\sigma)}=\rho, \quad0\leq\rho\leq+ \infty, $$

we call $F(s)$ of order *ρ* in the left half plane, where $\log^{+}x=\max\{\log x,0\}$. Furthermore, if $\rho\in (0,+\infty)$, the type of $F(s)$ is defined by

$$\limsup_{\sigma\rightarrow0^{-}}\frac{\log^{+}M_{u}(\sigma,F)}{(-\frac {1}{\sigma})^{\rho}}=T, \quad0\leq T\leq+\infty. $$

### Remark 1.1

However, if $\rho=0$ and $\rho=+\infty$, we cannot estimate the growth of such functions precisely by using the concept of type.

In 2012 and 2014, Luo and Kong [9, 10] investigated the growth of Laplace–Stieltjes transform converging on the whole plane and obtained the following.

### Theorem 1.1

(see [10])

*If the L*-*S transform* $F(s)\in L_{\infty}$ *and is of order* *ρ* ($0<\rho <\infty$), *then*

$$\rho=\limsup_{n\rightarrow+\infty}\frac{\lambda_{n}\log\lambda _{n}}{-\log A_{n}^{*}}. $$

### Theorem 1.2

(see [9])

*If the L*-*S transform* $F(s)\in L_{\infty}$, *then for* $p=1$, *we have*

$$\limsup_{\sigma\rightarrow+\infty}\frac{h(\log M_{u}(\sigma ,F))}{h(\sigma)}-1= \limsup_{n\rightarrow+\infty} \frac{h(\lambda_{n})}{h (-\frac {1}{\lambda_{n}}\log A_{n}^{*} )}, $$

*and for* $p=2,3,\ldots$ , *we have*

$$\begin{aligned} \limsup_{n\rightarrow+\infty}\frac{h(\lambda_{n})}{h (-\frac {1}{\lambda_{n}} \log A_{n}^{*} )}&\leq\limsup _{\sigma\rightarrow+\infty}\frac {h(\log M_{u}(\sigma,F))}{h(\sigma)} \\ &\leq\limsup_{n\rightarrow+\infty}\frac{h(\lambda_{n})}{h (-\frac{1}{\lambda_{n}}\log A_{n}^{*} )}+1, \end{aligned}$$

*where* $h(x)$ *satisfies the following conditions*:

(i) $h(x)$ *is defined on* $[a,+\infty)$ *and is positive*, *strictly increasing*, *differentiable and tends to* +∞ *as* $x\rightarrow+\infty$;
(ii) $\lim_{x\rightarrow+\infty}\frac {d(h(x))}{d(\log^{[p]}x)}=k\in(0,+\infty)$, $p\geq1$, $p\in\mathbb {N}^{+}$, *where* $\log^{[0]}x=x$, $\log^{[1]}x=\log x$, *and* $\log ^{[p]}x=\log(\log^{[p-1]}x)$.

In this paper, the first aim is to investigate the growth of analytic functions represented by Laplace–Stieltjes transforms with generalized order converging in the half plane, and we obtain some theorems about the generalized order $A_{n}^{*}$ and $\lambda_{n}$, which are improvements of the previous results given by Luo and Kong [9, 10]. To state our results, we first introduce the following notations and definitions.

Let Γ be a class of continuous increasing functions $\mathscr {A}$ such that $\mathscr{A}(x)\geq0$ for $x\geq x_{0}$, $\mathscr {A}(x)=\mathscr{A}(x_{0})$ for $x\leq x_{0}$ and on $[x_{0},+\infty)$ the function $\mathscr{A}$ increases to +∞; and $\Gamma^{0}$ be a class such that $\Gamma^{0}\subset\Gamma$ and $\mathscr {A}(x(1+o(1))=(1+o(1))\mathscr{A}(x)$ as $x\rightarrow+\infty$, for $\mathscr{A}\in\Gamma^{0}$; further, $\mathscr{A}\in\Gamma^{0i}$ if $\mathscr{A}\in\Gamma$ and for any $\eta>0$, $\mathscr{A}(\eta x)=(1+o(1))\mathscr{A}(x)$ as $x\rightarrow+\infty$. Obviously, it follows $\Gamma^{0i}\subset\Gamma^{0}$ and $h(x)\in\Gamma$.

### Definition 1.2

Let $F(s)\in L_{0}$ and $\mathscr{A}\in\Gamma$, $\mathscr{B}\in \Gamma$. If

$$\rho_{\mathscr{A}\mathscr{B}}(F)=\limsup_{\sigma\rightarrow 0^{-}}\frac{\mathscr{A}(\log M_{u}(\sigma,F))}{\mathscr{B}(-\frac {1}{\sigma})}, $$

then $\rho_{\mathscr{A}\mathscr{B}}$ is called generalized order of $F(s)$.

### Remark 1.2

Let $\mathscr{A}(x)=\log x$ and $\mathscr{B}=\log x$, then $\rho _{\mathscr{A}\mathscr{B}}(F)=\rho$.

### Remark 1.3

Let $\mathscr{A}(x)=\log_{p} x$ and $\mathscr{B}=\log_{q} x$, then $\rho_{\mathscr{A}\mathscr{B}}(F)=\rho(p,q)(F)$, where $\rho (p,q)(F)$ is the $(p,q)$-order of $F(s)$ (see [2]).

### Remark 1.4

Let $\mathscr{A}(x)=\log x$ and $\mathscr{B}=\log\log x$, then $\rho _{\mathscr{A}\mathscr{B}}(F)=\rho_{l}(F)$, where $\rho_{l}(F)$ is the logarithmic order of $F(s)$.

## Results and discussion

For generalized order of Laplace–Stieltjes transform (1), we obtain the following.

### Theorem 2.1

*Let* $F(s)\in L_{0}$, $\mathscr{A}\in\Gamma^{0i}$ *and* $\mathscr{B}\in \Gamma^{0i}$ *be continuously differentiable*, *and the function* $\mathscr{B}$ *increase more rapidly than* $\mathscr{A}$ *such that*, *for any constant* $\eta\in(0,+\infty )$,

8 $$ \frac{x}{\mathscr{B}^{-1}(\eta\mathscr{A}(x))}\rightarrow+\infty \quad(x_{0}\leq x \rightarrow+\infty ), $$

*and*

9 $$ \mathscr{A} \biggl(\frac{x}{\mathscr{B}^{-1}(\eta\mathscr {A}(x))} \biggr)=\bigl(1+o(1)\bigr) \mathscr{A}(x) \quad(x\rightarrow+\infty ). $$

*If*

10 $$ \lim_{\sigma\rightarrow0^{-}}\frac{\log M_{u}(\sigma,F)}{-\log (-\sigma)}=+\infty $$

*and*

$$\rho_{\mathscr{A}\mathscr{B}}(F)= \limsup_{\sigma\rightarrow 0^{-}}\frac{\mathscr{A}(\log M_{u}(\sigma,F))}{\mathscr{B}(-\frac {1}{\sigma})}, \quad 0\leq\rho_{\mathscr{A}\mathscr{B}}(F)\leq +\infty , $$

*then*

$$\rho_{\mathscr{A}\mathscr{B}}(F)=\limsup_{n\rightarrow+\infty }\frac{\mathscr{A}(\lambda_{n})}{\mathscr{B} (\frac{\lambda _{n}}{\log A_{n}^{*}} )}. $$

### Theorem 2.2

*Let* $F(s)\in L_{0}$, $\mathscr{A}\in\Gamma^{0i}$, *and* $\mathscr{B}\in \Gamma^{0i}$ *be continuously differentiable*, *and the function* $\mathscr{A}$ *increase more rapidly than* $\mathscr{B}$ *such that*, *for any constant* $\eta\in(0,+\infty )$,

11 $$ \frac{x}{\mathscr{A}^{-1}(\eta\mathscr{B}(x))}\uparrow+\infty \quad(x_{0}\leq x \rightarrow+\infty ), $$

*and*

12 $$ \mathscr{B} \biggl(\frac{x}{\mathscr{A}^{-1}(\eta\mathscr {B}(x))} \biggr)=\bigl(1+o(1)\bigr) \mathscr{B}(x) \quad(x\rightarrow+\infty ). $$

*If* $F(s)$ *satisfies* (10) *and*

$$\rho_{\mathscr{A}\mathscr{B}}(F)= \limsup_{\sigma\rightarrow 0^{-}}\frac{\mathscr{A}(\log M_{u}(\sigma,F))}{\mathscr{B}(-\frac {1}{\sigma})}, \quad 0\leq\rho_{\mathscr{A}\mathscr{B}}(F)\leq +\infty , $$

*then*

$$\rho_{\mathscr{A}\mathscr{B}}(F)=\limsup_{n\rightarrow+\infty }\frac{\mathscr{A}(\log A_{n}^{*})}{\mathscr{B} (\lambda_{n} )}. $$

If Laplace–Stieltjes transform (1) satisfies $A^{*}_{n}=0$ for $n\geq k+1$ and $A^{*}_{k}\neq0$, then $F(s)$ will be said to be an exponential polynomial of degree *k* usually denoted by $p_{k}$, *i.e.*, $p_{k}(s)=\int^{\lambda_{k}}_{0}\exp(sy)\,d\alpha(y)$. If we choose a suitable function $\alpha(y)$, the function $p_{k}(s)$ may be reduced to a polynomial in terms of $\exp(s\lambda_{i})$, that is, $\sum_{i=1}^{k}b_{i}\exp(s\lambda_{i})$. We denote $\Pi_{k}$ to be the class of all exponential polynomials of degree almost *k*, that is,

$$\Pi_{k}= \Biggl\{ \sum_{i=1}^{k} b_{i}\exp(s\lambda_{i}): (b_{1},b_{2}, \ldots ,b_{k})\in \mathbb{C}^{k} \Biggr\} . $$

For $F(s)\in\overline{L}_{\beta}$, $-\infty<\beta<0$, we denote by $E_{n}(F,\beta)$ the error in approximating the function $F(s)$ by exponential polynomials of degree *n* in uniform norm as

$$E_{n}(F,\beta)=\inf_{p\in\Pi_{n}}\Vert F-p \Vert_{\beta}, \quad n=1,2,\ldots, $$

where

$$\Vert F-p\Vert_{\beta}=\max_{-\infty< t< +\infty} \bigl\vert F(\beta +it)-p(\beta+it) \bigr\vert . $$

In 2017, Singhal and Srivastava [17] studied the approximation of Laplace–Stieltjes transforms of finite order converging on the whole plane and obtained the following theorem.

### Theorem 2.3

(see [17])

*If Laplace–Stieltjes transform* $F(s)\in L_{\infty}$ *and is of order* *ρ* ($0<\rho<\infty$) *and of type* *T*, *then for any real number* $-\infty<\beta<+\infty$, *we have*

$$\rho=\limsup_{n\rightarrow+\infty}\frac{\lambda_{n}\log\lambda _{n}}{-\log E_{n-1}(F,\beta)\exp(-\beta\lambda_{n})}=\limsup _{n\rightarrow+\infty}\frac{\lambda_{n}\log\lambda_{n}}{-\log E_{n-1}(F,\beta)} $$

*and*

$$T=\limsup_{n\rightarrow+\infty}\frac{\lambda_{n}}{\rho e}\bigl(E_{n-1}(F, \beta)\exp(-\beta\lambda_{n})\bigr)^{\frac{\rho}{\lambda_{n}}} =\limsup _{n\rightarrow+\infty}\frac{\lambda_{n}}{\rho\exp(\rho \beta+1)}\bigl(E_{n-1}(F,\beta) \bigr)^{\frac{\rho}{\lambda_{n}}}. $$

In the same year, the author and Kong [20] investigated the approximation of Laplace–Stieltjes transform $F(s)\in L_{0}$ with infinite order and obtained the following.

### Theorem 2.4

(see [20, Theorem 2.5])

*If the Laplace–Stieltjes transform* $F(s)\in L_{0}$ *and is of infinite order*, *if* $\lambda_{n}\sim\lambda_{n+1}$, *then for any real number* $-\infty<\beta<+\infty$, *then for any fixed real number* $-\infty<\alpha<0$, *we have*

13 $$ \limsup_{\sigma\rightarrow0^{-}}\frac{X(\log^{+}M_{u}(\sigma,F))}{\log (-\frac{1}{\sigma})}=\rho^{*} \quad \Longleftrightarrow\quad \limsup_{n\rightarrow\infty}\frac{X(\lambda_{n})}{\log^{+}\frac {\lambda_{n}}{\log^{+} [E_{n-1}(F,\alpha)\exp\{-\alpha\lambda _{n}\} ]}}=\rho^{*}, $$

*where* $0<\rho^{*}<\infty$, $X(\cdot)$-*order can be seen in* [20].

The second purpose of this paper is to study the approximation of Laplace–Stieltjes transform $F(s)\in L_{0}$ with generalized order, and our results are listed as follows.

### Theorem 2.5

*Let* $F(s)\in L_{0}$, $\mathscr{A}\in\Gamma^{0i}$, *and* $\mathscr{B}\in \Gamma^{0i}$ *be continuously differentiable satisfying* (8) *and* (9), *and let the function* $\mathscr{B}$ *increase more rapidly than* $\mathscr{A}$. *If* $F(s)$ *satisfies* (10) *and*

$$\rho_{\mathscr{A}\mathscr{B}}(F)= \limsup_{\sigma\rightarrow 0^{-}}\frac{\mathscr{A}(\log M_{u}(\sigma,F))}{\mathscr{B}(-\frac {1}{\sigma})}, \quad 0\leq\rho_{\mathscr{A}\mathscr{B}}(F)\leq +\infty , $$

*then for any real number* $-\infty <\beta<0$, *we have*

$$\rho_{\mathscr{A}\mathscr{B}}(F)=\limsup_{n\rightarrow+\infty }\frac{\mathscr{A}(\lambda_{n})}{\mathscr{B} (\frac{\lambda _{n}}{\log [E_{n-1}(F,\beta)\exp\{-\beta\lambda_{n}\} ]} )}. $$

### Theorem 2.6

*Under the assumptions of Theorem* 2.2, *then for any real number* $-\infty <\beta<0$, *we have*

$$\rho_{\mathscr{A}\mathscr{B}}(F)=\limsup_{n\rightarrow+\infty }\frac{\mathscr{A}(\log E_{n-1}(F,\beta))}{\mathscr{B} (\lambda_{n} )}. $$

## Conclusions

Regarding Theorems 2.1 and 2.2, the generalized order of Laplace–Stieltjes transforms are discussed by using the more abstract functions, and some related theorems among $\lambda_{n}$, $A_{n}^{*}$ and the generalized order are obtained. Moreover, we also investigate some properties of approximation on analytic functions defined by Laplace–Stieltjes transforms of generalized order. For the topic of the growth and approximation of Laplace–Stieltjes transforms of generalized order, it seems that this topic has never been treated before. Our theorems are generalization and improvement of the previous results given by Luo and Kong [9, 10], Singhal and Srivastava [17].

## Methods

To prove our results, we also need to give the following lemmas (see [16]).

Let $\Xi_{0}$ denote the set of positive unbounded functions *ϕ* on $(-\infty,0)$ such that the derivative $\phi'$ is positive, continuous, and increasing to +∞ on $(-\infty,0)$. Thus, if $\phi\in\Xi_{0}$, then $\phi(x)\rightarrow\zeta\geq0$ and $\phi '(x)\rightarrow0$ as $x\rightarrow-\infty$. Let *φ* be the inverse function of $\phi'$, then *φ* is continuous on $(0,+\infty)$ and increases to 0. Set $\phi\in\Xi_{0}$ and $\psi (x)=x-\frac{\phi(x)}{\phi'(x)}$. For $-\infty< x< x+\iota<0$, since $\phi'$ is increasing on $(-\infty,0)$, we have

$$\begin{aligned} \phi'(x)\phi(x+\iota)-\phi'(x+\iota)\phi(x)&< \phi'(x)\bigl[\phi (x+\iota)-\phi(x)\bigr] =\phi'(x) \int_{x}^{x+\iota}\phi'(t)\,dt \\ &< (x+\iota-x)\phi'(x)\phi'(x+\iota), \end{aligned}$$

that is,

$$\psi(x)=x-\frac{\phi(x)}{\phi'(x)}< x+\iota-\frac{\phi(x+\iota )}{\phi'(x+\iota)}=\psi(x+\iota). $$

Thus, it means that *ψ* is an increasing function on $(-\infty,0)$.

Next, we will prove that $\psi(x)\rightarrow0$ as $x\rightarrow0$, that is, there is no constant $\eta<0$ such that $\psi(x)\leq\eta$ for all $x\in(-\infty,0)$. Assume that there exist two constants *η*, $K_{1}$ such that $\psi(x)\leq\eta$ for all $x\in(-\infty,0)$ and $\eta< K_{1}<0$. Since *ψ* is an increasing function and $\psi (x)< x<0$, then it follows $\frac{\phi'(x)}{\phi(x)}\leq\frac{1}{x-\eta}$ for $K_{1}\leq x<0$. Thus, it follows

$$\begin{aligned} \log\phi(x)&=\log\phi(K_{1})+ \int_{K_{1}}^{x}\frac{\phi'(t)}{\phi (t)}\,dt\leq\log \phi(K_{1})+ \int_{K_{1}}^{x}\frac{1}{t-\eta}\,dt \\ &=\log\phi(K_{1})+\log\frac{x-\eta}{K_{1}-\eta}. \end{aligned}$$

Hence $\phi(x)\leq\phi(K_{1})\frac{x-\eta}{K_{1}-\eta}$. In view of $\phi'(x)\rightarrow+\infty$ ($x\rightarrow0$), we get a contradiction. Thus, it follows $\psi(x)\rightarrow0$ as $x\rightarrow0$.

Besides, let $\psi^{-1}$ be the inverse function of *ψ*. Then $\psi^{-1}$ is an increasing function on $(-\infty, 0)$ and $\phi '(\psi^{-1}(\sigma))$ increases to +∞ on $(-\infty,0)$.

### Lemma 4.1

*Let* $\phi\in\Xi_{0}$, *then the conclusion that* $\log\mu(\sigma ,F)\leq\phi(\sigma)$ *for any* $\sigma\in(-\infty,0)$ *holds if and only if* $\log A_{n}^{*}\leq-\lambda_{n}\psi(\varphi(\lambda_{n}))$ *for all* $n\geq0$.

### Proof

Suppose that $\log\mu(\sigma,F)\leq\phi(\sigma)$ for any $\sigma \in(-\infty,0)$, then $\log A_{n}^{*}\leq\phi(\sigma)-\sigma\lambda _{n}$ for all $n>0$ and $\sigma\in(-\infty,0)$. Thus, take $\sigma =\varphi(\lambda_{n})$, it follows for all $n\geq0$ that

$$\begin{aligned} \log A_{n}^{*}&\leq\phi\bigl(\varphi(\lambda_{n})\bigr)- \lambda_{n}\varphi(\lambda_{n}) =-\lambda_{n} \biggl(\varphi(\lambda_{n})-\frac{\phi(\varphi(\lambda _{n}))}{\phi'(\varphi(\lambda_{n}))} \biggr) =- \lambda_{n}\psi\bigl(\varphi(\lambda_{n})\bigr). \end{aligned}$$

On the contrary, assume that $\log A_{n}^{*}\leq-\lambda_{n}\psi(\varphi (\lambda_{n}))$ for all $n\geq0$. Since, for any $\sigma<0$ and $x<0$,

$$(\sigma-x)\phi'(x)\leq \int_{x}^{\sigma}\phi'(t)\,dt=\phi(\sigma)- \phi(x), $$

then it follows

$$\begin{aligned} \log\mu(\sigma,F)&\leq\max\bigl\{ -\lambda_{n}\psi\bigl(\varphi( \lambda _{n})\bigr)+\lambda_{n}\sigma: n\geq0\bigr\} \leq \max\bigl\{ -t\psi\bigl(\varphi(t)\bigr)+t\sigma: t\geq0\bigr\} \\ &=\max\bigl\{ -\phi'(x)\psi(x)+\sigma+\phi'(x): x>- \infty\bigr\} \\ &=\max\bigl\{ (\sigma-x)\phi'(x)+\phi(x): x>-\infty\bigr\} =\phi( \sigma). \end{aligned}$$

Therefore, this completes the proof of Lemma 4.1. □

### Lemma 4.2

*If the L*-*S transform* $F(s)\in L_{\infty}$, *then for any* *σ* ($-\infty <\sigma<0$) *and* *ε* (>0), *we have*

$$\frac{1}{p}\mu(\sigma,F)\leq M_{u}(\sigma,F)\leq C\mu\bigl((1- \varepsilon )\sigma,F\bigr)\frac{1}{-\sigma}, $$

*where* $p>2$ *and* *C* (≠0) *are constants*.

### Proof

We will adapt the method as in Yu [26] and Kong and Hong [5]. Set

$$I(x;\sigma+it)= \int_{0}^{x}\exp\bigl\{ (\sigma+it)y\bigr\} \,d \alpha(y). $$

In view of (5), there exists a positive number *ξ* satisfying $0<\lambda_{n+1}-\lambda_{n}\leq\xi$ ($n=1,2,3,\ldots$). Thus, it yields $e^{-\xi\sigma}<\frac{p}{2}$ for *σ* sufficiently close to 0^−^, where $p>2$ is a constant. For $x>\lambda_{n}$, it follows

$$\begin{aligned} \int_{\lambda_{n}}^{x}\exp\{ity\}\,d\alpha(y)&= \int_{\lambda_{n}}^{x}\exp\{ -\sigma y\}d_{y}I(y; \sigma+it) \\ &=I(y;\sigma+it)\exp\{-\sigma y\} \vert_{\lambda_{n}}^{x}+\sigma \int _{\lambda_{n}}^{x}\exp\{-\sigma y\}I(y;\sigma+it)\,dy. \end{aligned}$$

Then, for any $\sigma<0$ and any $x\in(\lambda_{n},\lambda_{n+1}]$, it yields

$$\begin{aligned} \biggl\vert \int_{\lambda_{n}}^{x}\exp\{ity\}\,d\alpha(y) \biggr\vert & \leq M_{u}(\sigma,F)\bigl[\exp\{-x\sigma\}+\exp\{-\sigma \lambda_{n}\}+ \bigl\vert \exp\{-x\sigma\}-\exp\{-\sigma \lambda_{n}\} \bigr\vert \bigr] \\ &\leq2 M_{u}(\sigma,F)\exp\bigl\{ -(\lambda_{n}+\xi)\sigma \bigr\} \leq pM_{u}(\sigma,F)\exp\{-\lambda_{n}\sigma\}, \end{aligned}$$

which implies

14 $$ \frac{1}{p}\mu(\sigma,F)\leq M_{u}(\sigma,F). $$

On the other hand, for any $x>0$, it follows that there exists a positive integer $n\in\mathbb{N}_{+}$ such that $\lambda_{n}< x\leq \lambda_{n+1}$. Thus, it follows

$$\int_{0}^{x}\exp\bigl\{ (\sigma+it)y\bigr\} \,d \alpha(y)=\sum_{k=1}^{n-1} \int _{\lambda_{k}}^{\lambda_{k+1}}\exp\bigl\{ (\sigma+it)y\bigr\} \,d \alpha(y) + \int_{\lambda_{n}}^{x}\exp\bigl\{ (\sigma+it)y\bigr\} \,d \alpha(y). $$

Set $I_{k}(x;it)=\int_{\lambda_{n}}^{x}\exp\{ity\}\,d\alpha(y)$ ($\lambda _{k}< x\leq\lambda_{k+1}$), then for any real number *t* and $\sigma <0$, it follows $|I_{k}(x;it)|\leq A_{k}^{*}\leq\mu(\sigma,F)e^{-\lambda _{k}\sigma}$. Thus, for any $\varepsilon\in(0,1)$ and $\sigma<0$, it yields $|I_{k}(x;it)|\leq\mu((1-\varepsilon)\sigma,F)e^{-\lambda _{k}(1-\varepsilon)\sigma}$ and

$$\begin{aligned} \int_{0}^{x}\exp\bigl\{ (\sigma+it)y\bigr\} \,d \alpha(y)&=\sum_{k=1}^{n-1}\biggl[\exp\{ \lambda_{k+1}\sigma\}I_{k}(\lambda_{k+1};it)-\sigma \int_{\lambda _{k}}^{\lambda_{k+1}}\exp\{\sigma y\}I_{k}(y;it) \,dy\biggr] \\ &\quad{}+\exp\{x\sigma\}I_{n}(x;it)-\sigma \int_{\lambda_{n}}^{x}\exp\{\sigma y\}I_{n}(y;it) \,dy. \end{aligned}$$

Hence, we can deduce

$$\begin{aligned} & \biggl\vert \int_{0}^{x}\exp\bigl\{ (\sigma+it)y\bigr\} \,d \alpha(y) \biggr\vert \\ &\quad \leq \sum_{k=1}^{n-1}\mu\bigl((1- \varepsilon)\sigma,F\bigr)\exp\bigl\{ -\lambda _{k}(1-\varepsilon)\sigma \bigr\} \bigl(\exp\{\lambda_{k+1}\sigma\}+ \bigl\vert \exp\{\lambda _{k+1}\sigma\}-\exp\{\lambda_{k}\sigma\} \bigr\vert \bigr) \\ &\qquad {} +\mu\bigl((1-\varepsilon)\sigma,F\bigr)\exp\bigl\{ -\lambda_{n}(1- \varepsilon )\sigma\bigr\} \bigl(\exp\{x\sigma\}+ \bigl\vert \exp\{x\sigma\}- \exp\{\lambda _{n}\sigma\} \bigr\vert \bigr) \\ &\quad =\mu\bigl((1-\varepsilon)\sigma,F\bigr)\sum_{k=1}^{+\infty } \exp\{\lambda _{k}\varepsilon\sigma\}. \end{aligned}$$

In view of (5), for the above *ε*, there exists $N_{1}\in N_{+}$ such that, for any $n>N_{1}$, we have $\lambda_{n} >\frac {n}{D+\varepsilon}$. Hence it follows for $\sigma\rightarrow0^{-}$ that

15 $$ \sum_{k=1}^{+\infty}\exp\{ \lambda_{k}\varepsilon\sigma\}\leq\sum_{k=1}^{N_{1}} \exp\{\lambda_{k}\varepsilon\sigma\}+ \sum_{k=N_{1}+1}^{+\infty } \exp \biggl\{ k\frac{\varepsilon\sigma }{D+\varepsilon} \biggr\} < C \frac{1}{-\sigma}, $$

where *C* is a constant on *ε* and (5). Therefore, this lemma is proved from (14) and (15). □

### Proofs of Theorems 2.1 and 2.2

#### The proof of Theorem 2.1

Suppose that $\rho:=\rho_{\mathscr{A}\mathscr{B}}(F)<+\infty $ and

$$\vartheta=\limsup_{n\rightarrow+\infty}\frac{\mathscr{A}(\lambda _{n})}{\mathscr{B} (\frac{\lambda_{n}}{\log A_{n}^{*}} )}. $$

In view of the definition of generalized order and Lemma 4.2, for any $\varepsilon>0$, there exists a constant $\sigma_{0}<0$ such that, for all $0>\sigma>\sigma_{0}$,

$$ \log\mu(\sigma,F)\leq\mathscr{A}^{-1} \biggl((\rho+\varepsilon ) \mathscr{B} \biggl(-\frac{1}{\sigma} \biggr) \biggr)+\log p, $$

that is,

16 $$ \log A_{n}^{*}\leq\mathscr{A}^{-1} \biggl(( \rho+\varepsilon)\mathscr {B} \biggl(-\frac{1}{\sigma} \biggr) \biggr)- \lambda_{n}\sigma+\log p, \quad n\geq0. $$

Choosing

$$-\frac{1}{\sigma}=\mathscr{B}^{-1} \biggl(\frac{1}{\rho+\varepsilon } \mathscr{A} \biggl(\frac{\lambda_{n}}{\mathscr{B}^{-1} (\frac {\mathscr{A}(\lambda_{n})}{\rho+\varepsilon} )} \biggr) \biggr), $$

we conclude from (9) and (16) that

$$\begin{aligned} \log A_{n}^{*}&\leq\frac{\lambda_{n}}{\mathscr{B}^{-1} (\frac{\mathscr{A}(\lambda_{n})}{\rho+\varepsilon} )}+\frac {\lambda_{n}}{\mathscr{B}^{-1} (\frac{1}{\rho+\varepsilon} \mathscr{A} (\frac{\lambda_{n}}{\mathscr{B}^{-1} (\frac {\mathscr{A}(\lambda_{n})}{\rho+\varepsilon} )} ) )}+\log p \\ &=\frac{(1+o(1))\lambda_{n}}{\mathscr{B}^{-1} ((1+o(1))\frac {\mathscr{A}(\lambda_{n})}{\rho+\varepsilon} )},\quad \mbox{as } n\rightarrow+\infty , \end{aligned}$$

which implies

17 $$ \mathscr{A}(\lambda_{n})\leq(\rho+\varepsilon) \mathscr{B} \biggl(\frac{(1+o(1))\lambda_{n}}{\log A_{n}^{*}} \biggr), \quad\mbox{as } n\rightarrow+\infty . $$

Since $\mathscr{A}\in\Gamma^{0i}$, $\mathscr{B}\in\Gamma^{0i}$ and let $\varepsilon\rightarrow0^{+}$, we can conclude from (17) that $\vartheta\leq\rho$.

Assume $\vartheta< \rho$, then we can choose a constant $\rho_{1}$ such that $\vartheta<\rho_{1}<\rho$. Since $\mathscr{B}^{-1} (\frac {\mathscr{A}(x)}{\rho_{1}} )$ is an increasing function, then there exists a positive integer $n_{0}$ such that, for $n\geq n_{0}$,

18 $$ \log A_{n}^{*}\leq\frac{\lambda_{n}}{\mathscr{B}^{-1} (\frac {\mathscr{A}(\lambda_{n})}{\rho_{1}} )}\leq \int_{\lambda _{n_{0}}}^{\lambda_{n}}\frac{1}{\mathscr{B}^{-1} (\frac{\mathscr {A}(t)}{\rho_{1}} )}\,dt+K_{1}, $$

where here and further $K_{j}$ is a constant.

Since $\phi\in\Xi_{0}$, and let

$$\phi(\sigma)= \int_{-\frac{1}{\sigma_{0}}}^{-\frac{1}{\sigma}}\frac {\mathscr{A}^{-1}(\rho_{1}\mathscr{B}(t))}{t^{2}}\,dt+K_{2} \quad \mbox{for } 0>\sigma\geq\sigma_{0}. $$

Then it follows

$$\begin{aligned} &\phi'(\sigma)=\mathscr{A}^{-1}\biggl(\rho_{1} \mathscr{B}\biggl(-\frac{1}{\sigma }\biggr)\biggr), \qquad \varphi( \lambda_{n})=-\frac{1}{\mathscr{B}^{-1} (\frac{\mathscr{A}(\lambda_{n})}{\rho_{1}} )}, \\ &\bigl[\lambda_{n}\psi\bigl(\varphi(\lambda_{n})\bigr) \bigr]'=\bigl[\lambda_{n}\varphi(\lambda _{n})-\phi\bigl(\varphi(\lambda_{n})\bigr) \bigr]'=\varphi(\lambda_{n}), \end{aligned} $$

and

19 $$ -\lambda_{n}\psi\bigl(\varphi(\lambda_{n}) \bigr)=- \int_{\lambda_{n_{0}}}^{\lambda _{n}}\varphi(t)\,dt+K_{2}= \int_{\lambda_{n_{0}}}^{\lambda_{n}}\frac {1}{\mathscr{B}^{-1} (\frac{\mathscr{A}(t)}{\rho_{1}} )}\,dt+K_{2}. $$

Thus, in view of (18) and (19), it follows

20 $$\begin{aligned} \log\mu(\sigma,F)&\leq\phi(\sigma)= \int_{-\frac {1}{\sigma_{0}}}^{-\frac{1}{\sigma}}\frac{\mathscr{A}^{-1}(\rho _{1}\mathscr{B}(t))}{t^{2}}\,dt+K_{2} \\ &\leq\mathscr{A}^{-1} \biggl(\rho_{1}\mathscr{B} \biggl(-\frac {1}{\sigma} \biggr) \biggr) \int_{-\frac{1}{\sigma_{1}}}^{-\frac {1}{\sigma}}\frac{dt}{t^{2}}+K_{3} \\ &\leq-\sigma_{1} \mathscr{A}^{-1} \biggl( \rho_{1}\mathscr{B} \biggl(-\frac{1}{\sigma} \biggr) \biggr)+K_{3}. \end{aligned}$$

Since $\mathscr{A}\in\Gamma^{0i}$, in view of (10), (20) and by applying Lemma 4.2, we can deduce $\rho_{\mathscr {A}\mathscr{B}}(F)\leq\rho_{1}$, which implies a contradiction with $\rho_{\mathscr{A}\mathscr {B}}(F)>\rho_{1}$. Hence $\vartheta=\rho_{\mathscr{A}\mathscr{B}}(F)$.

If $\rho_{\mathscr{A}\mathscr{B}}(F)=+\infty $, by using the same argument as above, it is easy to prove that the conclusion is true. Therefore, this completes the proof of Theorem 2.1.

#### The proof of Theorem 2.2

Suppose that $\rho:=\rho_{\mathscr{A}\mathscr{B}}(F)<+\infty $ and

$$\vartheta_{1}=\limsup_{n\rightarrow+\infty}\frac{\mathscr{A}(\log A_{n}^{*})}{\mathscr{B} (\lambda_{n} )}. $$

In view of the definition of generalized order and Lemma 4.2, for any $\varepsilon>0$, there exists a constant $\sigma_{0}<0$ such that, for all $0>\sigma>\sigma_{0}$,

$$ \log\mu(\sigma,F)\leq\mathscr{A}^{-1} \biggl((\rho+\varepsilon ) \mathscr{B} \biggl(-\frac{1}{\sigma} \biggr) \biggr)+\log p, $$

that is,

21 $$ \log A_{n}^{*}\leq\mathscr{A}^{-1} \biggl(( \rho+\varepsilon)\mathscr {B} \biggl(-\frac{1}{\sigma} \biggr) \biggr)- \lambda_{n}\sigma+\log p, \quad n\geq0. $$

Choosing $-\frac{1}{\sigma}=\lambda_{n}$, we conclude from (21) that

22 $$\begin{aligned} \log A_{n}^{*}&\leq\mathscr{A}^{-1}\bigl(( \rho+\varepsilon)\mathscr {B}(\lambda_{n})\bigr)+1+\log p \\ &\leq \bigl(1+o(1)\bigr)\mathscr{A}^{-1}\bigl((\rho +\varepsilon)\mathscr{B}( \lambda_{n})\bigr),\quad \mbox{as } n\rightarrow +\infty . \end{aligned}$$

Since $\mathscr{A}\in\Gamma^{0i}$, $\mathscr{B}\in\Gamma^{0i}$ and let $\varepsilon\rightarrow0^{+}$, we can conclude from (22) that $\vartheta_{1}\leq\rho$.

Assume $\vartheta_{1}< \rho$, then we can choose a constant $\rho_{2}$ such that $\vartheta_{1}<\rho_{2}<\rho$. It means that there exists a positive integer $n_{0}$ such that, for $n\geq n_{0}$,

$$\log A_{n}^{*}\leq\mathscr{A}^{-1}\bigl(\rho_{2} \mathscr{B}(\lambda_{n})\bigr), $$

that is,

23 $$ \log\mu(\sigma,F)\leq\max\bigl\{ \mathscr{A}^{-1}\bigl( \rho_{2}\mathscr {B}(\lambda_{n})\bigr)+\lambda_{n} \sigma: n\geq n_{0}\bigr\} +K_{5}. $$

In view of (10), the following equation

$$\mathscr{A}^{-1}\bigl(\rho_{2}\mathscr{B}(t)\bigr)+t\sigma=0 $$

has a unique solution $t_{1}:=t(\sigma)$ such that $t_{1}\uparrow+\infty $ as $\sigma\rightarrow0^{-}$, and for $t\geq t_{1}$ we can deduce that $\mathscr{A}^{-1}(\rho_{2}\mathscr{B}(t))+t\sigma\leq0$. Hence, it follows

24 $$\begin{aligned} \log\mu(\sigma,F)&\leq\max\bigl\{ \mathscr{A}^{-1}\bigl(\rho _{2}\mathscr{B}(t)\bigr)+t\sigma: t_{0}\leq t\leq t_{1}\bigr\} +K_{6} \\ &\leq\mathscr{A}^{-1}\bigl(\rho_{2}\mathscr{B}(t_{1}) \bigr)+K_{6}. \end{aligned}$$

In view of

$$-\frac{1}{\sigma}=\frac{t_{1}}{\mathscr{A}^{-1}(\rho_{2}\mathscr{B}(t_{1}))}, $$

it follows from (12) that

25 $$ \mathscr{B} \biggl(-\frac{1}{\sigma} \biggr)=\mathscr{B} \biggl(\frac {t_{1}}{\mathscr{A}^{-1}(\rho_{2}\mathscr{B}(t_{1}))} \biggr)=\bigl(1+o(1)\bigr)\mathscr{B}(t_{1}), \quad\sigma\rightarrow0^{-}. $$

Hence, we can deduce from (24) and (25) that

$$\log\mu(\sigma,F)\leq\mathscr{A}^{-1} \biggl(\rho _{2} \bigl(1+o(1)\bigr)\mathscr{B} \biggl(-\frac{1}{\sigma} \biggr) \biggr),\quad \mbox{as } \sigma\rightarrow0^{-}, $$

which implies $\rho_{\mathscr{A}\mathscr{B}}(F)\leq\rho_{2}<\rho _{\mathscr{A}\mathscr{B}}(F)$ by combining Lemma 4.2 and (10), a contradiction. Therefore, $\vartheta_{1}=\rho_{\mathscr {A}\mathscr{B}}(F)$.

If $\rho_{\mathscr{A}\mathscr{B}}(F)=+\infty $, by using the same argument as above, it is easy to prove that the conclusion is true. Therefore, this completes the proof of Theorem 2.2.

### Proofs of Theorems 2.5 and 2.6

#### The proof of Theorem 2.5

Suppose that $\rho:=\rho_{\mathscr{A}\mathscr{B}}(F)<+\infty $ and

$$\vartheta_{3}=\limsup_{n\rightarrow+\infty}\frac{\mathscr {A}(\lambda_{n})}{\mathscr{B} (\frac{\lambda_{n}}{\log [E_{n-1}(F,\beta)\exp\{-\beta\lambda_{n}\} ]} )}. $$

In view of the definition of generalized order and Lemma 4.2, for any $\varepsilon>0$, there exists a constant $\sigma_{0}<0$ such that, for all $0>\sigma>\sigma_{0}$,

26 $$ \log M_{u}(\sigma,F)\leq\mathscr{A}^{-1} \biggl((\rho+\varepsilon )\mathscr{B} \biggl(-\frac{1}{\sigma} \biggr) \biggr). $$

Since $F(s)\in L_{0}$, and for any constant *β* ($-\infty<\beta<0$), then $F(s)\in \overline{L}_{\beta}$. Hence, for $\beta<\sigma<0$ and $p_{k}\in\Pi _{k}$, it follows

27 $$\begin{aligned} E_{k}(F,\beta) &\leq\Vert F-p_{k} \Vert_{\beta}\leq \bigl\vert F(\beta +it)-p_{k}(\beta+it) \bigr\vert \\ &\leq \biggl\vert \int_{0}^{+\infty}\exp\{sy\}\,d\alpha(y)- \int _{0}^{\lambda_{k}}\exp\{sy\}\,d\alpha(y) \biggr\vert = \biggl\vert \int_{\lambda_{k}}^{\infty}\exp\{sy\}\,d\alpha(y) \biggr\vert . \end{aligned}$$

Let

$$I_{j+k}(b;it)= \int_{\lambda_{j+k}}^{b}\exp\{ity\}\,d\alpha(y) \quad ( \lambda_{j+k}< b\leq\lambda_{j+k+1}), $$

then $|I_{j+k}(b;it)|\leq A_{j+k}^{*}$. In view of

$$\biggl\vert \int_{\lambda_{k}}^{\infty}\exp\bigl\{ (\beta+it)y\bigr\} \,d\alpha (y) \biggr\vert =\lim_{b\rightarrow+\infty} \biggl\vert \int_{\lambda _{k}}^{b}\exp\bigl\{ (\beta+it)y\bigr\} \,d \alpha(y) \biggr\vert , $$

where $-\infty <\beta<0$, hence

$$\begin{aligned} & \biggl\vert \int_{\lambda_{k}}^{b}\exp\bigl\{ (\beta+it)y\bigr\} \,d\alpha (y) \biggr\vert \\ &\quad = \Biggl\vert \sum_{j=k}^{n+k-1} \int_{\lambda_{j}}^{\lambda _{j+1}}\exp\{\beta y\}d_{y}I_{j}(y;it)+ \int_{\lambda_{n+k}}^{b}\exp\{\beta y\}d_{y}I_{n+k}(y;it) \Biggr\vert \\ &\quad = \Biggl\vert \Biggl[\sum_{j=k}^{n+k-1}e^{\lambda_{j+1}\beta }I_{j}( \lambda_{j+1};it)-\beta \int_{\lambda_{j}}^{\lambda _{j+1}}e^{\beta y}I_{j}(y;it) \,dy \Biggr] \\ &\qquad{}+e^{\beta b}I_{n+k}(b;it)-\beta \int_{\lambda _{n+k}}^{b}e^{\beta y}I_{j}(y;it) \,dy \Biggr\vert \\ &\quad \leq \sum_{j=k}^{n+k-1} \bigl[A_{j}^{*}e^{\lambda_{j+1}\beta }+A_{j}^{*} \bigl(e^{\lambda_{j+1}\beta}-e^{\lambda_{j}\beta}\bigr) \bigr] +2e^{\beta\lambda_{n+k+1}}A_{n+k}^{*}-e^{\beta\lambda _{n+k}}A_{n+k}^{*} \\ &\quad \leq 2\sum_{j=k}^{n+k} A_{n}^{*}e^{\lambda_{n+1}\beta}. \end{aligned}$$

Therefore, we conclude

28 $$ \biggl\vert \int_{\lambda_{k}}^{\infty}\exp\bigl\{ (\beta+it)y\bigr\} \,d\alpha (y) \biggr\vert \leq2\sum_{n=k}^{+\infty}A_{n}^{*} \exp\{\beta\lambda _{n+1}\}, \quad \mbox{as } n\rightarrow+\infty . $$

In view of Lemma 4.2, it follows $A_{n}^{*}\leq p M_{u}(\sigma ,F)e^{-\sigma\lambda_{n}}$. So, for any *σ* ($\beta<\sigma<0$), it yields from (27) and (28) that

29 $$ E_{n}(F,\beta)\leq2\sum_{k=n+1}^{\infty}A_{k-1}^{*}\exp\{\beta\lambda _{k}\}\leq2pM_{u}( \sigma,F)\sum_{k=n+1}^{\infty}\exp\bigl\{ (\beta- \sigma )\lambda_{k}\bigr\} . $$

In view of (5), we can choose $h'$ ($0< h'< h$) such that $(\lambda_{n+1} -\lambda_{n})\geq h'$ for $n\geq0$. Then, for $\sigma\geq \frac{\beta}{2}$, it follows from (29) that

$$\begin{aligned} E_{n}(F,\beta) &\leq M_{u}(\sigma,F)\exp\bigl\{ \lambda_{n+1}(\beta-\sigma)\bigr\} \sum_{k=n+1}^{\infty}\exp\bigl\{ (\lambda_{k}-\lambda_{n+1}) (\beta-\sigma)\bigr\} \\ &\leq M_{u}(\sigma,F)\exp\bigl\{ \lambda_{n+1}(\beta-\sigma) \bigr\} \exp \biggl\{ -\frac{\beta}{2}h'(n+1) \biggr\} \sum _{k=n+1}^{\infty}\exp \biggl\{ \frac{\beta}{2}h'k \biggr\} \\ &=M_{u}(\sigma,F)\exp\bigl\{ \lambda_{n+1}(\beta-\sigma)\bigr\} \biggl(1-\exp\biggl\{ \frac{\beta}{2}h'\biggr\} \biggr)^{-1}, \end{aligned}$$

that is,

30 $$ E_{n-1}(F,\beta)\leq KM_{u}(\sigma,F)\exp\bigl\{ \lambda_{n}(\beta-\sigma )\bigr\} , $$

where *K* is a constant. Hence, it follows from (26) and (30) that

31 $$ \log\bigl[E_{n-1}(F,\beta)\exp\{-\beta \lambda_{n}\}\bigr]\leq\mathscr {A}^{-1} \biggl((\rho+ \varepsilon)\mathscr{B} \biggl(-\frac{1}{\sigma } \biggr) \biggr)- \lambda_{n}\sigma+\log K, \quad n\geq0. $$

Let

$$-\frac{1}{\sigma}=\mathscr{B}^{-1} \biggl(\frac{1}{\rho+\varepsilon } \mathscr{A} \biggl(\frac{\lambda_{n}}{\mathscr{B}^{-1} (\frac {\mathscr{A}(\lambda_{n})}{\rho+\varepsilon} )} \biggr) \biggr), $$

we conclude from (9) and (31) that

$$\begin{aligned} \log\bigl[E_{n-1}(F,\beta)\exp\{-\beta\lambda_{n}\} \bigr]&\leq\frac {\lambda_{n}}{\mathscr{B}^{-1} (\frac{\mathscr{A}(\lambda _{n})}{\rho+\varepsilon} )}+\frac{\lambda_{n}}{\mathscr {B}^{-1} (\frac{1}{\rho+\varepsilon} \mathscr{A} (\frac{\lambda_{n}}{\mathscr{B}^{-1} (\frac {\mathscr{A}(\lambda_{n})}{\rho+\varepsilon} )} ) )}+\log K \\ &=\frac{(1+o(1))\lambda_{n}}{\mathscr{B}^{-1} ((1+o(1))\frac {\mathscr{A}(\lambda_{n})}{\rho+\varepsilon} )}, \quad\mbox{as } n\rightarrow+\infty , \end{aligned}$$

which implies

32 $$ \mathscr{A}(\lambda_{n})\leq(\rho+\varepsilon) \mathscr{B} \biggl(\frac{(1+o(1))\lambda_{n}}{\log[E_{n-1}(F,\beta)\exp\{-\beta \lambda_{n}\}]} \biggr), \quad \mbox{as } n\rightarrow+ \infty . $$

Since $\mathscr{A}\in\Gamma^{0i}$, $\mathscr{B}\in\Gamma^{0i}$ and let $\varepsilon\rightarrow0^{+}$, we can conclude from (32) that $\vartheta_{3}\leq\rho$.

Assume $\vartheta_{3}< \rho$, then we can choose a constant $\rho_{3}$ such that $\vartheta_{3}<\rho_{3}<\rho$. Since $\mathscr{B}^{-1} (\frac {\mathscr{A}(x)}{\rho_{3}} )$ is an increasing function, then there exists a positive integer $n_{0}$ such that, for $n\geq n_{0}$,

33 $$ \log\bigl[E_{n-1}(F,\beta)\exp\{-\beta \lambda_{n}\}\bigr]\leq\frac{\lambda _{n}}{\mathscr{B}^{-1} (\frac{\mathscr{A}(\lambda_{n})}{\rho _{3}} )}\leq \int_{\lambda_{n_{0}}}^{\lambda_{n}}\frac {1}{\mathscr{B}^{-1} (\frac{\mathscr{A}(t)}{\rho_{3}} )}\,dt+K_{1}. $$

For any $\beta<0$, then there exists $p_{1}\in\Pi_{n-1}$ such that

34 $$ \Vert F-p_{1} \Vert \leq2E_{n-1}(F,\beta). $$

And since

$$\begin{aligned} A_{n}^{*}\exp\{\beta\lambda_{n}\}& =\sup _{\lambda_{n}< x\leq\lambda_{n+1},-\infty< t< +\infty } \biggl\vert \int_{\lambda_{n}}^{x}\exp\{ity\}\,d\alpha(y) \biggr\vert \exp\{\beta\lambda_{n}\} \\ &\leq\sup_{\lambda_{n}< x\leq\lambda_{n+1},-\infty< t< +\infty } \biggl\vert \int_{\lambda_{n}}^{x}\exp\bigl\{ (\beta+it)y\bigr\} \,d \alpha(y) \biggr\vert \\ &\leq\sup_{-\infty< t< +\infty} \biggl\vert \int_{\lambda _{n}}^{\infty}\exp\bigl\{ (\beta+it)y\bigr\} \,d \alpha(y) \biggr\vert , \end{aligned}$$

thus, for any $p\in\Pi_{n-1}$, it follows

35 $$ A_{n}^{*}\exp\{\beta\lambda_{n}\}\leq \bigl\vert F(\beta+it)-p(\beta +it) \bigr\vert \leq \Vert F-p \Vert _{\beta}. $$

Hence, for any $\beta<0$ and $F(s)\in L_{0}$, it follows from (34) and (35) that

36 $$ A_{n}^{*}\leq2E_{n-1}(F,\beta)\exp\{-\beta \lambda_{n}\}. $$

Hence, (18) follows from (33) and (36).

Since $\phi\in\Xi_{0}$, and let

$$\phi(\sigma)= \int_{-\frac{1}{\sigma_{0}}}^{-\frac{1}{\sigma}}\frac {\mathscr{A}^{-1}(\rho_{1}\mathscr{B}(t))}{t^{2}}\,dt+K_{2} \quad \mbox{for } 0>\sigma\geq\sigma_{0}, $$

and

$$\varphi(\lambda_{n})=-\frac{1}{\mathscr{B}^{-1} (\frac{\mathscr {A}(\lambda_{n})}{\rho_{1}} )}. $$

By using the same argument as in the proof of Theorem 2.1, we conclude

37 $$\begin{aligned} \log\mu(\sigma,F)&\leq\phi(\sigma)= \int_{-\frac {1}{\sigma_{0}}}^{-\frac{1}{\sigma}}\frac{\mathscr{A}^{-1}(\rho _{1}\mathscr{B}(t))}{t^{2}}\,dt+K_{2} \\ &\leq-\sigma_{1} \mathscr{A}^{-1} \biggl( \rho_{1}\mathscr{B} \biggl(-\frac{1}{\sigma} \biggr) \biggr)+K_{3}. \end{aligned}$$

Since $\mathscr{A}\in\Gamma^{0i}$, in view of (10), (37) and by applying Lemma 4.2, we can deduce $\rho_{\mathscr {A}\mathscr{B}}(F)\leq\rho_{3}$, which implies a contradiction with $\rho_{\mathscr{A}\mathscr {B}}(F)>\rho_{1}$. Hence $\vartheta_{3}=\rho_{\mathscr{A}\mathscr{B}}(F)$.

If $\rho_{\mathscr{A}\mathscr{B}}(F)=+\infty $, by using the same argument as above, it is easy to prove that the conclusion is true. Therefore, this completes the proof of Theorem 2.5.

#### The proof of Theorem 2.6

By combining the arguments as in the proofs of Theorems 2.2 and 2.5, we can easily prove the conclusion of Theorem 2.6.
